# Half-Brain Delineation for Prediction of Radiation-Induced Temporal Lobe Injury in Nasopharyngeal Carcinoma Receiving Intensity-Modulated Radiotherapy

**DOI:** 10.3389/fonc.2021.599942

**Published:** 2021-04-01

**Authors:** Qing-Hua Du, Yi-Xiu Gan, Ren-Sheng Wang, Wen-Qi Liu, Jian Li, Fei-Fei Liang, Xiang-De Li, Hui-Jun Zhu, Xue Ou, Qiu-Lu Zhong, Dan-Jing Luo, Zhi-Peng Zhu, Shang-Yong Zhu

**Affiliations:** ^1^ Department of Radiation Oncology, Second Affiliated Hospital of Guangxi Medical University, Nanning, China; ^2^ Department of Radiation Oncology, First Affiliated Hospital of Guangxi Medical University, Nanning, China; ^3^ Department of Medical Ultrasound, First Affiliated Hospital of Guangxi Medical University, Nanning, China

**Keywords:** temporal lobe injury, half-brain, delineation, nasopharyngeal carcinoma, prediction

## Abstract

**Purpose:**

To investigate the role of half-brain delineation in the prediction of radiation-induced temporal lobe injury (TLI) in nasopharyngeal carcinoma (NPC) receiving intensity-modulated radiotherapy (IMRT).

**Methods and Materials:**

A total of 220 NPC cases treated with IMRT and concurrent platinum-based chemotherapy were retrospectively analyzed. Dosimetric parameters of temporal lobes, half-brains, and brains included maximum dose (D_max_), doses covering certain volume (D_V_) from 0.03 to 20 cc and absolute volumes receiving specific dose (V_D_) from 40 to 80 Gy. Inter-structure variability was assessed by coefficients of variation (CV) and paired samples *t*-tests. Receiver operating characteristic curve (ROC) and Youden index were used for screening dosimetric parameters to predict TLI. Dose/volume response curve was calculated using the logistic dose/volume response model.

**Results:**

CVs of brains, left/right half-brains, and left/right temporal lobes were 9.72%, 9.96%, 9.77%, 27.85%, and 28.34%, respectively. Each D_V_ in temporal lobe was significantly smaller than that in half-brain (P < 0.001), and the reduction ranged from 3.10% to 45.98%. The area under the curve (AUC) of D_V_ and V_D_ showed an “increase-maximum-decline” behavior with a peak as the volume or dose increased. The maximal AUCs of D_V_s in brain, half-brain and temporal lobe were 0.808 (D_2cc_), 0.828 (D_1.2cc_) and 0.806 (D_0.6cc_), respectively, and the maximal AUCs of V_D_s were 0.818 (D_75Gy_), 0.834 (V_72Gy_) and 0.814 (V_70Gy_), respectively. The cutoffs of V_70Gy_ (0.86 cc), V_71Gy_ (0.72 cc), V_72Gy_ (0.60 cc), and V_73Gy_ (0.45 cc) in half-brain had better Youden index. TD5/5 and TD50/5 of D_1.2cc_ were 58.7 and 80.0 Gy, respectively. The probability of TLI was higher than >13% when V_72Gy_>0 cc, and equal to 50% when V_72Gy_ = 7.66 cc.

**Conclusion:**

Half-brain delineation is a convenient and stable method which could reduce contouring variation and could be used in NPC patients. D_1.2cc_ and V_72Gy_ of half-brain are feasible for TLI prediction model. The dose below 70 Gy may be relatively safe for half-brain. The cutoff points of V_70–73Gy_ could be considered when the high dose is inevitable.

## Introduction

Radiation-induced temporal lobe injury (TLI) is a serious complication for nasopharyngeal carcinoma (NPC), which has profound effects on quality of life (1). Understanding the probability of developing temporal lobe injury is an important requirement of radiotherapy for NPC patients. The quantitative analysis of normal tissue effects in the clinic (QUANTEC) review showed that for conventional fractionation with doses ≤2 Gy, a 5% risk of symptomatic radiation brain necrosis is predicted at an equivalent dose of 72 Gy (2). In 2019, an international guideline on dose prioritization and acceptance criteria for NPC was developed (3). The final temporal lobe recommendation of the panel was to aim for a D_0.03cc_ planning risk volume (PRV) dose ≤ 65 Gy for T1–2 tumors and ≤ 70 Gy for T3–4 tumors. However, the optimal dose/volume predictors for TLI still vary in different studies. A study by Sun et al. (4) reported that a D_0.5cc_ of 69 Gy might be the dose tolerance of the temporal lobe. Other studies suggested different dose equivalents of 58 Gy (D_1cc_) (5), 60.3 Gy (D_2cc_) (6), 62.8 Gy (D_1cc_) (7), and 69 Gy (D_max_ at 2 Gy per fraction) (8) for a 5% probability of developing temporal lobe injury at 5 years. Considering the long incubation period and few cases of radiation temporal lobe injury, more practical data are needed to support the accurate dose limit.

Accurate delineation of temporal lobe is another important requirement. Significant inter-observer variation in delineation of target volumes or normal organs has been demonstrated (9–11), which might also occur in delineation of temporal lobe (12, 13). In order to collect accurate data for TLI prediction, temporal lobes were re-contoured in some studies (6, 8, 14). Sun et al. (15) provided a contouring recommendation for temporal lobe, which reduced the delineation divergence. Temporal lobe contouring can be standardized through effective implementation of a temporal lobe contouring protocol and atlas, but it requires continuous and extensive training for beginners (13). On the other hand, magnetic resonance imaging (MRI) fusion, which makes the temporal lobe clearer, is not performed for every case.

Brain is a structure clearly defined by international guidelines (16). Surrounded by a clear skull bone, the brain could be easily delineated with little disagreement, and the automatic segmentation of brain is more feasible. However, brain is rarely contoured in NPC patients. Half-brain (left and right half-brain, corresponding to left and right temporal lobe) might be a simple substitute for temporal lobe considering that: 1) only a small high-dose volume of temporal lobe is used for TLI prediction; 2) the high-dose volume is always concentrated in temporal pole. Therefore, the small high-dose volume is present simultaneously in half-brain. Even the whole brain might predict TLI independently. In order to confirm the role of half-brain delineation in TLI prediction, this study compared the dosimetric parameters of temporal lobe and half-brain, and assessed the predictive ability of brain, half-brain and temporal lobe for TLI.

## Materials and Methods

### Patient Selection

From January 2009 to May 2015, 220 NPC patients treated with IMRT and concurrent platinum-based chemotherapy at the First Affiliated Hospital of Guangxi Medical University were retrospectively reviewed (**Table 1**). Patients were followed every 3 months in the first 2 years and every 6 months during the next 3 years, and then annually thereafter. The median follow-up time of was 69.3 months (range, 61.1–120.8 months). The incidence of TLI was 34.5%, and the median latency was 39.3 months (range, 1.4–78.7 months).

**Table 1:** Basic characteristics for 220 patients.

Items	No.	Injury	Non-injury	P
Gender				0.829
Male	166	58 (76.3%)	108 (75.0%)	
Female	54	18 (23.7%)	36 (25.0%)	
Age				0.559
>50	50	19 (25.0%)	31(21.5%)	
≤50	170	57 (75.0%)	113 (78.5%)	
Diabetes				0.938
Yes	9	3 (3.9%)	6 (4.2%)	
No	215	73 (96.1%)	138 (95.8%)	
Hypertension				0.896
Yes	11	4 (5.3%)	7 (4.9%)	
No	215	72 (94.7%)	137 (95.1%)	
T stage*				<0.001
T1	0	0 (0%)	0 (0%)	
T2	25	2 (2.6%)	23 (16.0%)	
T3	86	15 (19.7%)	71 (49.3%)	
T4	109	59 (77.6%)	50 (34.7%)	
TLI	76			
Left	26			
Right	30			
Both	20			
Fraction				
30	62			
31	103			
32	24			
33	31			

P value was derived from the univariable association analyses between each of the clinical variables and injury status. For binary variables, a chi-square test was used.

*When T stage and the following dosimetric parameters were analyzed together in multivariate analysis, T stage was removed (P > 0.05).

### Radiation Therapy and Structure Delineation

**Figure 1:**
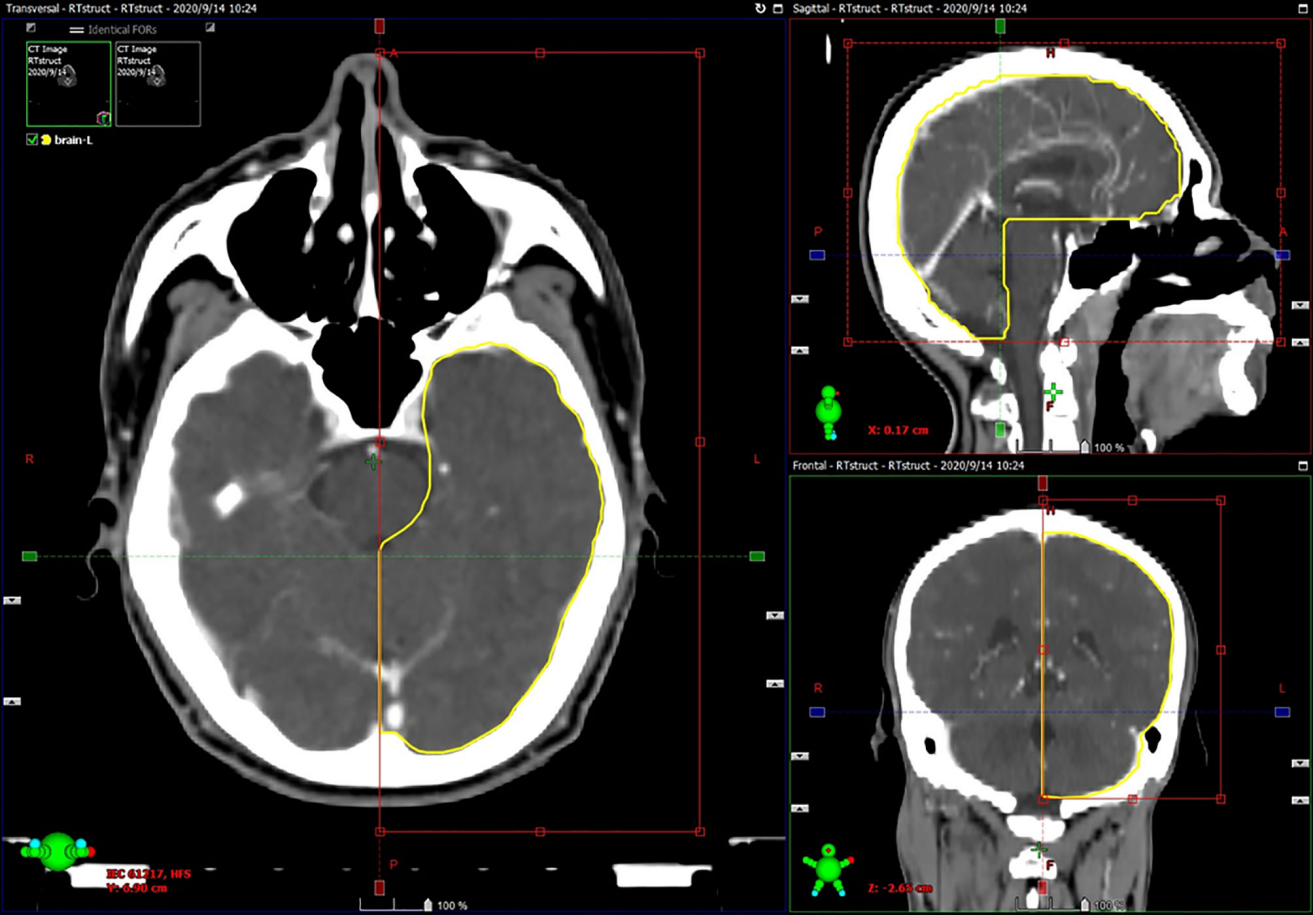
Example of half-brain delineation: automatic segmentation was limited to one half of the brain according to the brain midline on coronal image, and errors were corrected by manual contouring.

A neck and shoulder thermoplastic mask was used to fix the patients. Radiation planning was designed and optimized using inverse treatment planning system (software version: Pinnacle 9.8 and Varian Eclipse 9.8), at least 5 isocentric fields being set up. The prescribed dose was 68 to 72 Gy to the planning target volume (PTV) of gross tumor volume (GTV), 60 to 64 Gy to the PTV of high-risk clinical target volume (CTV), and 50 to 54 Gy to the PTV of low-risk CTV. The doses for each critical organ were limited, as described in the Radiation Therapy Oncology Group 02-25 protocol (eg, point, 65 Gy and 1% volume, 60 Gy for temporal lobes) (7). When doses exceeded limits inevitably, they were accepted by consensus and adequate communication with patients. All patients received full-course IMRT in 30 to 33 fractions, one fraction daily over 5 days per week. The brain was contoured primarily by automatic segmentation (errors were corrected by manual contouring) in all cases as only the pure brain parenchyma was considered, excluding the cavernous sinuses, the brainstem, optic chiasm, optical tract, pituitary gland, mammillary bodies, and Meckel’s caves (16, 17). For the purpose of this study, the brain was divided into left half-brain and right half-brain according to the brain midline on coronal image (**Figure 1**). The temporal lobes contoured (similar to the method 1 in Sun’s study (15) but the basal ganglia and insula were excluded) by the radiotherapists previously were directly adopted.

### Toxicity Endpoints

The MRI images were reviewed by two radiologists and a radiation oncologist, and disagreements were resolved by consensus. Diagnostic criteria for TLI were as follows (6): (a) white matter lesions, defined as areas of finger-like lesions of increased signal intensity on T2-weighted images; (b) contrast-enhanced lesions, defined as lesions with or without necrosis on post-contrast T1-weighted images with heterogeneous signal abnormalities on T2-weighted images; (c) cysts, round or oval well defined lesions of very high signal intensity on T2-weighted images with a thin or imperceptible wall as previously reported.

### Dosimetric Parameters

The dose-volume histograms (DVH) were exported from the treatment planning system. Dosimetric parameters included maximum dose (D_max_), doses covering certain volume (D_V_) from 0.03 to 20 cc and absolute volumes receiving specific dose (V_D_) from 40 Gy to 80 Gy. Equivalent dose in 2 Gy (EQD_2_) was calculated by linear quadratic model (EQD_2_=D_x_(d_x_+α∕β)/(2+α∕β)) (18) with an α∕β ratio of 3 Gy (17).

### Statistical Analysis

SPSS 19.0 was used for statistical analysis. The variations in delineation of temporal lobe, half-brain, and brain were assessed by Coefficients of variation (CV). D_V_s in half-brain and temporal lobe were compared using paired samples *t*-tests. Receiver operating characteristic curve (ROC) was used for screening dosimetric parameters to predict TLI. The prediction ability was assessed by the area under the curve (AUC) and Youden index. Dose and volume response curves were calculated with the nonlinear regression model using the logistic dose/volume response model (19) as *P(X)* =1/(1+exp (-*b*_0_-*b*_1_ *X*)), where *X* is the value of D_V_ or V_D_.

## Results

### Descriptive Statistics of Volumes, D_V_ and V_D_

Mean volumes of brains, left half-brains, right half-brains, left temporal lobes, and right temporal lobes were 1303.84 ± 126.78 cc, 640.35 ± 63.81 cc, 659.61 ± 64.47 cc, 66.50 ± 18.52 cc, and 70.39 ± 19.95 cc, respectively. CVs of them were 9.72%, 9.96%, 9.77%, 27.85%, and 28.34%, respectively. Paired samples *t*-tests showed that each D_V_ in temporal lobe was significantly smaller than that in half-brain (P < 0.001), and the reduction ranged from 3.10% to 45.98% (**Table 2**). Pearson correlation analysis showed that all the D_V_s in each structure were associated with each other significantly, as well as V_D_s (P < 0.001).

**Table 2:** Comparison of D_V_s in half-brain and temporal lobe.

Variable	Mean (Gy)		Difference (Gy)	Reduction (%)	P
	half-brain	temporal lobe			
D_max_	78.37 ± 8.86	75.94 ± 8.78	2.43 ± 4.85	3.10 ± 6.19	<0.001
D_0.03cc_	76.30 ± 9.18	73.72 ± 9.01	2.58 ± 4.88	3.38 ± 6.40	<0.001
D_0.5cc_	70.72 ± 10.03	67.51 ± 10.19	3.20 ± 5.05	4.53 ± 7.14	<0.001
D_0.6cc_	70.02 ± 10.16	66.73 ± 10.34	3.29 ± 5.06	4.70 ± 7.23	<0.001
D_0.7cc_	69.37 ± 10.28	66.01 ± 10.48	3.36 ± 5.05	4.84 ± 7.28	<0.001
D_0.8cc_	68.77 ± 10.39	65.34 ± 10.61	3.43 ± 5.03	4.99 ± 7.31	<0.001
D_0.9cc_	68.20 ± 10.49	64.70 ± 10.73	3.49 ± 5.03	5.12 ± 7.38	<0.001
D_1cc_	67.66 ± 10.58	64.10 ± 10.84	3.56 ± 5.03	5.30 ± 7.49	<0.001
D_1.1cc_	67.15 ± 10.67	63.52 ± 10.93	3.63 ± 5.03	5.37 ± 7.43	<0.001
D_1.2cc_	66.92 ± 10.71	62.97 ± 11.03	3.95 ± 5.08	5.90 ± 7.59	<0.001
D_1.3cc_	66.21 ± 10.83	62.44 ± 11.12	3.77 ± 5.07	5.69 ± 7.66	<0.001
D_1.4cc_	65.77 ± 10.91	61.93 ± 11.19	3.84 ± 5.08	5.84 ± 7.72	<0.001
D_1.5cc_	65.34 ± 10.97	61.43 ± 11.27	3.91 ± 5.10	5.98 ± 7.81	<0.001
D_2cc_	63.40 ± 11.22	59.13 ± 11.57	4.27 ± 5.18	6.73 ± 8.17	<0.001
D_3cc_	60.28 ± 11.53	55.24 ± 12.06	5.04 ± 5.40	8.36 ± 8.96	<0.001
D_4cc_	57.82 ± 11.69	51.94 ± 12.46	5.88 ± 5.64	10.17 ± 9.76	<0.001
D_5cc_	55.78 ± 11.76	49.02 ± 12.77	6.76 ± 5.78	12.12 ± 10.36	<0.001
D_10cc_	48.78 ± 11.67	37.48 ± 13.84	11.3 ± 6.38	23.16 ± 13.08	<0.001
D_20cc_	40.93 ± 11.19	22.11 ± 13.27	18.82 ± 7.05	45.98 ± 17.22	<0.001

### Variations of ROC in Different Structures

**Figure 2:**
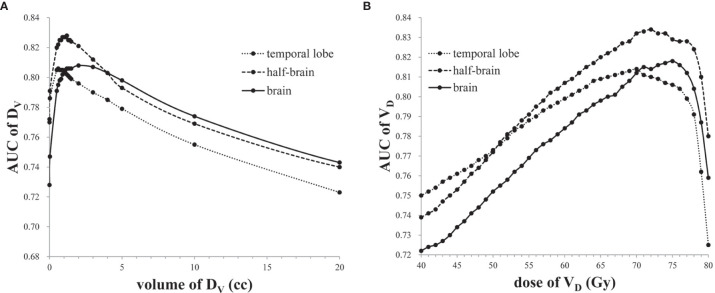
**(A)** The AUCs of D_V_s in temporal lobe, half-brain, and brain. **(B)** The AUCs of V_D_s in temporal lobe, half-brain, and brain.

The AUCs of D_V_s and V_D_s showed an “increase -maximum-decline” behavior with a peak as the volume or dose increased (**Figure 2**). The maximal AUCs of D_V_s in brain, half-brain, and temporal lobe were 0.808 (D_2.0cc_), 0.828 (D_1.2cc_) and 0.806 (D_0.6cc_), respectively. The maximal AUCs of V_D_s in brain, half-brain, and temporal lobe were 0.818 (D_75Gy_), 0.834 (V_72Gy_), and 0.814 (V_70Gy_), respectively. The cutoff of V_72Gy_ (0.60 cc) in half-brain showed the largest Youden index (0.568). Further analysis of all the dose/volume points showed that the cutoffs of V_70Gy_ (0.86 cc), V_71Gy_ (0.72 cc), and V_73Gy_ (0.45 cc) in half-brain also had the same or better Youden index (**Table 3**).

**Table 3:** The best AUCs and cutoffs in temporal lobe, half-brain, and brain.

	AUC	95% CI		Cutoff			
		Lower	Upper	Value	Sensitivity	Specificity	Youden index
Temporal lobe							
D_0.6cc_	0.806	0.757	0.854	68.99 Gy	0.854	0.695	0.549
V_70Gy_	0.814	0.769	0.860	0.45 cc	0.865	0.686	0.551
Half-brain							
D_1.2cc_	0.828	0.783	0.872	67.49 Gy	0.885	0.651	0.536
V_72Gy_	0.834	0.790	0.877	0.60 cc	0.896	0.672	0.568
V_70Gy_	0.832	0.788	0.875	0.86 cc	0.896	0.672	0.568
V_71Gy_	0.833	0.790	0.876	0.72 cc	0.896	0.672	0.568
V_73Gy_	0.832	0.788	0.876	0.48 cc	0.896	0.677	0.573
brain							
D_2cc_	0.808	0.748	0.868	75.67 Gy	0.684	0.833	0.517
V_75Gy_	0.818	0.760	0.876	2.22 cc	0.684	0.833	0.517

### Dose/Volume Response Model

**Figure 3:**
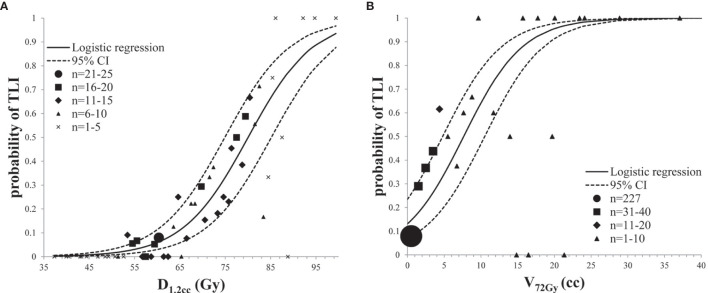
Prediction models for radiation-induced TLI: **(A)** dose response analysis of D_1.2cc_ in half-brain; **(B)** volume response analysis of V_72Gy_ in half-brain.

Because of significant collinearity of dosimetric parameters, multivariate analysis was not considered. D_1.2cc_ and V_72Gy_ in half-brain were enrolled for dose/volume response model due to better AUC. Independent logistic regression analysis was performed with each dosimetric factor (**Table 4**). Two dose/volume response curves were generated and demonstrated an increasing effect probability with increasing dose/volume (**Figure 3**). TD5/5 and TD50/5 of D_1.2cc_ were 58.7 Gy (95% CI: 53.6–63.8) and 80.0 Gy (95% CI: 74.9–85.2), respectively. The probability of TLI was higher than 13% when V_72Gy_>0 cc (95% CI: 0–2.87), and equal to 50% when V_72Gy_ = 7.66 cc (95% CI: 4.79–10.52).

**Table 4:** Logistic regression analysis results of D_1.2cc_ and V_72Gy_ in half-brain.

	B	SE	Wald	Sig	Exp (B)
D_1.2cc_	0.138	0.016	73.640	<0.001	1.148
Constant	−11.045	1.192	85.786	<0.001	0.000
V_72Gy_	0.247	0.040	38.126	<0.001	1.281
Constant	−1.891	0.156	147.320	<0.001	0.151

## Discussion

The dosimetric parameters are the major variables that influence the development of radiation-induced TLI. Other suggested risk factors include chemotherapy use, radiation technique, and T stage (5–7, 20). However, T stage is correlated with dose and prescription. When T stage and dose are analyzed together, T stage would be removed by analysis model (21). In order to reduce the influence of chemotherapy use and radiotherapy technique, only the patients who treated with IMRT and concurrent platinum-based chemotherapy were included. In this study, when T stage and the following dosimetric parameters were analyzed together in multivariate analysis, T stage was removed. Therefore, the only independent risk factor was dosimetric parameters in this study.

Because of the long latency period (6, 20, 22, 23), the incidence of radiation-induced TLI may be underestimated if the follow-up is insufficient. Studies have shown an incidence between 0% and 40.3% in NPC patients (6, 20, 24–26). In this study, the higher incidence of TLI may be related to follow-up bias and advanced T-stage (symptomatic patients were more likely to complete follow-up). However, incidence should be estimated based on dosimetric parameters. Predictive models attempt to provide a versatile and objective estimate of a patient’s probability of developing treatment related complications (17). Marks et al. considered that the information provided by QUANTEC is generally not ideal for most of organs, and care must be taken to apply it correctly in the clinic (27). The ideal information might require substantial, more comparable and reliable supporting data. To ensure the accuracy of prediction, target volumes should be highly consistent and repeatable.

Whether the parahippocampal, hippocampus, basal ganglia, and insula were included in temporal lobe was debatable before Sun’s recommendation (15). There is little disagreement regarding image segmentation of the entire brain, and little movement occurs (2). Brain could be easily contoured by rapidly evolving automatic and robust segmentation technology (28–30). Large volume of temporal lobe is contoured in NPC patients, but only a small hot spot volume about 1 cc is used for prediction in most of the studies (3). The high-dose regions are mainly distributed in bilateral temporal pole in NPC patients, and the intermediate structure, such as brainstem, optic chiasm, optical tract, pituitary gland, and mammillary bodies are excluded from brain delineation (16). Thus the half-brain delineation might replace the temporal lobe delineation in NPC patients, considering that the high-dose regions of two half-brains rarely overlap. In this study, CVs of both brains and half-brains were less than 10%, but the CVs of temporal lobes by manual contouring without rigidly standardized training were close to 30%, indicating that brain and half-brain are more stable structures with less contouring variation.

Compared to D_V_s in half-brain, D_V_s in temporal lobe reduced by less than 5% when the volume was less than 0.8 cc, indicating that the hot spot, which is the common predictor, is likely included in both half-brain and temporal lobe although the CV of temporal lobes is large. Therefore, half-brain might be a simple substitute for the temporal lobe. In this study, the maximal AUC in half-brain was better than that in temporal lobe. In addition to the difference in temporal lobe delineation, the possible reason is that parahippocampal and hippocampus were not included in temporal lobe in this study. Therefore, some volumes with high/sub-high dose were excluded, which might affect the prediction ability. To avoid this, parahippocampal and hippocampus should be included in temporal lobe, which is also suggested in Sun’s recommendation (15). While in extreme cases, the highest dose of 1 cc may present outside the temporal lobe, it is not a bad thing that it could predict other brain injury.

Considering that brain structure is defined by international guidelines (16), the predictive ability of brain was also assessed in this study. The results showed that the AUC in brain was lower than that in half-brain. That is probably because the brain including more dispersed hot spots (bilateral dose deposition) could not predict TLI accurately. In this study, the optimal dosimetric parameters and limits of three structures were different, which indicated that target volume should have a high consistency to ensure the reliability of the prediction model.

The AUCs of D_V_s/V_D_s in each structure showed an “increase-maximum-decline” behavior with a peak as the volume or dose increased, indicating that the dose of extremely small hot spot volume, such as D_max_ and D_0.03cc_, might not be a reasonable parameter of TLI prediction model. The possible reason is that the small volume of the hot spot is easily influenced by contouring, and easily manipulated by the treatment planner, or by the optimization software. Nevertheless, D_max_ or D_0.03cc_ might be used as a dose monitoring point of tolerated dose. Zhou et al. found that V_D_ at a dose of ≥70 Gy was found with the highest odds ratio (23). In this study, the V_D_ points of V_73Gy_ = 0.45 cc, V_72Gy_ = 0.60 cc, V_71Gy_ = 0.72 cc, and V_70Gy_ = 0.86 cc had better Youden index, indicating that 70 Gy may be a sensitive and specific cutoff dose. Therefore, D_max_/D_0.03cc_ < 70 Gy might be relatively safe, which is also suggested by international guideline (3).

However, the best cutoff does not mean the best probability prediction parameter. Stable and representative volumes are important to overall predictive capacity. In this study, D_1.2cc_ and V_72Gy_ in half-brain were enrolled for dose/volume response model due to better AUCs. TD5/5 and TD50/5 of D_1.2cc_ were 58.7 and 80.0 Gy, respectively. The probability of TLI was higher than 13% when V_72Gy_>0 cc, and equal to 50% when V_72Gy_=7.66 cc. Considering the difference of reference volume, the AUC, TD5/5 and TD50/5 are roughly similar to previous studies (4, 6, 8), indicating that half-brain delineation is feasible for TLI prediction model.

There are several limitations in this study. Firstly, the temporal lobe may have better predictive power after standardized training toward observers, which was not involved in this study. Second, the fraction is not uniform, which may influence the predictive ability. Thirdly, the half-brain delineation method is limited to NPC patients, and new errors may be introduced comparing with only delineating temporal lobe. Finally, the application of half-brain delineation needs to be confirmed in more studies, especially in multi-center studies.

## Conclusion

Half-brain delineation is a convenient and stable method which could reduce contouring variation and could be used in TLI prediction model in NPC patients. D_1.2cc_ and V_72Gy_ of half-brain are feasible for TLI prediction model. TD5/5 and TD50/5 of D_1.2cc_ are 58.7 Gy and 80.0 Gy, respectively. The probability of TLI is higher than 13% when V_72Gy_>0 cc, and equal to 50% when V_72Gy_=7.66 cc. The dose below 70 Gy may be relatively safe for half-brain. The cutoff points of V_73Gy_=0.45 cc, V_72Gy_=0.60 cc, V_71Gy_=0.72 cc, and V_70Gy_=0.86 cc could be considered when the high dose of half-brain is inevitable.

## Data Availability Statement

The original contributions presented in the study are included in the article/supplementary material. Further inquiries can be directed to the corresponding author.

## Ethics Statement

The studies involving human participants were reviewed and approved by the review board of the Second Affiliated Hospital of Guangxi Medical University. Written informed consent for participation was not required for this study in accordance with the national legislation and the institutional requirements.

## Author Contributions

S-YZ and W-QL participated in the study concept and design. JL, F-FL, X-DL, H-JZ, XO, Q-LZ, D-JL, and Z-PZ participated in the acquisition of data. Q-HD and Y-XG participated in the analysis and interpretation of data. Q-HD participated in the statistical analysis. Q-HD and R-SW participated in the drafting of the manuscript. All authors contributed to the article and approved the submitted version.

## Funding

This work was supported by Science Foundation of Guangxi Zhuang Autonomous Region Health and Family Planning Commission [award Z20181010& Z20181011] and Science Foundation of Second Affiliated Hospital of Guangxi Medical University [award EFYKY2020008].

## Conflict of Interest

The authors declare that the research was conducted in the absence of any commercial or financial relationships that could be construed as a potential conflict of interest.
